# Clinical manifestations and genetic analysis of a newborn with Arboleda−Tham syndrome

**DOI:** 10.3389/fgene.2022.990098

**Published:** 2022-10-25

**Authors:** Feng Zeng, Yue Yang, Zhaohui Xu, Ziwen Wang, Huan Ke, Jianhong Zhang, Tongtong Dong, Wenming Yang, Jiuxiang Wang

**Affiliations:** ^1^ Department of Neonatology, Xuancheng Central Hospital, Xuancheng, Anhui, China; ^2^ Department of Neurology, The First Affiliated Hospital of Anhui University of Chinese Medicine, Hefei, Anhui, China; ^3^ Department of Paediatrics, The First Affiliated Hospital of Anhui University of Chinese Medicine, Hefei, Anhui, China; ^4^ Graduate School, Anhui University of Chinese Medicine, Hefei, Anhui, China; ^5^ Nursing Department, Xuancheng Central Hospital, Xuancheng, Anhui, China; ^6^ Experimental Center of Clinical Research, The First Affiliated Hospital of Anhui University of Chinese Medicine, Hefei, Anhui, China

**Keywords:** Arboleda−Tham syndrome, newborn, asphyxia, KAT6A, mouse growth factor

## Abstract

Arboleda−Tham syndrome (ARTHS) is a rare disorder first characterized in 2015 and is caused by mutations in lysine (K) acetyltransferase 6A (KAT6A, a.k.a. MOZ, MYST3). Its clinical symptoms have rarely been reported in newborns from birth up to the first few months after birth. In this study, a newborn was diagnosed with ARTHS based on the clinical symptoms and a mutation c.3937G>A (p.Asp1313Asn) in KAT6A. The clinical manifestations, diagnosis, and treatment of the newborn with ARTHS were recorded during follow-up observations. The main symptoms of the proband at birth were asphyxia, involuntary breathing, low muscle tone, early feeding, movement difficulties, weak crying, weakened muscle tone of the limbs, and embrace reflex, and facial features were not obvious at birth. There was obvious developmental delay, as well as hypotonic and oro-intestinal problems in the first few months after birth. Mouse growth factor was used to nourish the brain nerves, and touching, kneading the back, passive movements of the limbs, and audio−visual stimulation were used for rehabilitation. We hope that this study expands the phenotypic spectrum of this syndrome to newborns and the library of KAT6A mutations that lead to ARTHS. Consequently, the data can be used as a basis for genetic counseling and in clinical and prenatal diagnosis for ARTHS prevention.

## Introduction

Arboleda−Tham syndrome (MIM: 616268), also known as KAT6A syndrome, is a rare disorder mainly characterized by developmental delay, intellectual disability, and distinct facial features. It was first characterized in 2015, and initial descriptions included 10 affected cases (Tham et al., 2015). To date, about 90 patients have been reported, the youngest of which was diagnosed when 8 months old (Arboleda et al., 2015; Tham et al., 2015; Bscch et al., 2019; Lin et al., 2020). Clinical symptoms have rarely been reported in newborns from birth up to the first few months after birth.

KAT6A protein is a lysine acetyltransferase belonging to the MYST family of acetyltransferases. It comprises 2,004 amino acids, a double PHD (plant homeodomain) zinc finger domain, a highly conserved MYST domain, a glutamate/aspartate-rich region, and a serine/methionine-rich region in the C-terminal region (Champagne et al., 1999; Champagne et al., 2001). The PHD zinc finger domains interact with histone H3 substrate and are required for the nuclear localization of chromatin (Ali et al., 2012; Dreveny et al., 2014). The MYST domain, which includes the acetyl-CoA-binding motif, enhances acetylation by binding to DNA through the zinc and helix-turn-helix motifs (Holbert et al., 2007). The glutamate/aspartate-rich region, also known as the NEMM (N-terminal part of Enok, MOZ, or MORF) domain, is conserved in *Drosophila* Enok and is important for the nuclear localization of KAT6A (Yang and Ullah, 2007). The serine/methionine-rich region possesses transcription activation activity (Kitabayashi et al., 2001).

KAT6A is an epigenetic modifier. It is a acetyltransferases acylate histone H3 and nonhistone proteins and is involved in numerous biological and developmental processes such as regulation of transcription, maintenance of hematopoietic and neural stem cells, hematopoietic cell differentiation, cell cycle, and mitosis. It interacts with BRPF1/2/3 (bromodomain–PHD finger protein), ING5 (inhibitor of growth 5), and MEAF6 (MYST/Esa1-associated factor 6) to form a tetrameric complex to acetylate lysine residues on histone H3 tails such as H3K9, H3K23, and H3K14 (Ullah et al., 2008; Qiu et al., 2012; Voss and Thomas, 2018). These histone modifications are associated with transcriptionally active genes *Tbx1*, *Tbx5T*, *Dlx5* locus, and *PI3K YAP* (Voss et al., 2012; Vanyai et al., 2019a; Vanyai et al., 2019b). *KAT6A* homozygous deletion mice (*KAT6A* ^−/−^ mice) have ventricular septal defects, and more than 50% of individuals with inactivating mutations in KAT6A or its paralog KAT6B present with ventricular septal defects and other congenital heart abnormalities (Vanyai et al., 2015; Bscch et al., 2019).

Currently, *KAT6A* is the only known ARTHS disease–causing gene, and more than 50 mutations have been identified in approximately 90 cases. This case report describes the clinical symptoms of a newborn with ARTHS from birth up to the first 17 weeks, and the treatment and genetic tests conducted.

## Materials and methods

### Case

This study was conducted according to the Declaration of Helsinki and was approved by the ethics committee of the Xuancheng Central Hospital (2021010). Written informed consent was obtained from the parents of the newborn. The newborn was born at the Xuancheng Central Hospital.

### Exome capture, sequencing, and variation detection

Genomic DNA was isolated from the peripheral venous blood for WES to screen for the candidate causative mutation using IDT xGen Exome Research Panel and NovaSeq 6000 system (Illumina). The reads were mapped to the human reference genome (hg38) with BWA, SAMtools, and Picard software, obtaining 3.2 G bases mapped to the target exome regions with a mean depth of 183.99 times, and 99.71% of the exome was covered at least ten times. The GATK program was applied to perform base quality score recalibration, indel realignment, duplicate removal, SNP and INDEL discovery, and genotype scoring using the standard filtering parameters according to GATK. In total, 250,875 genetic variants were identified and annotated by ANNOVAR. The pathogenic variants were screened according to the ACMG classification guidelines and the clinical phenotype of the patients: 1) screening for variants and non-synonymous mutations in the exon region. 2) Screening for variants that are absent in the ExAC_EAS, ExAC_ALL, 1000 Genomes, and gnomAD databases, or minor allele frequency (MAF) < 0.01. 3) Evaluating the variants by reference to the dbSNP, OMIM, HGMD, ClinVar, and other databases. 4) Predicting the effect of variants on the protein function by SIFT, PolyPhen-2, LRT, MutationTaster, and FATHMM. Sanger sequencing was conducted to further confirm *KAT6A.* The control group comprised 100 DNA samples from healthy individuals.

## Results

### Clinical features of infant from birth to when 17 weeks old and treatment

The infant was born at 37 + 1 weeks by cesarean with Apgar scores of 5/7 at 1 and 5 min. He weighed 2.3 kg, was 47 cm long, and had a cranial circumference of 33 cm. The infant was asphyxiated for 31 min; he did not cry and had no suck, swallow, or gag reflex. His limbs had reduced muscle tone, undescended testicles, and a flat skull. His cardiac malformations showed an atrial septal defect of 3.0 mm, patent ductus arteriosus, and moderate pulmonary hypertension (Table 1).

**TABLE 1:** Clinical characteristics of the case.

Age	1 day	4 weeks	10 weeks	17 weeks
Weight	2.3 kg	2.7 kg	4.2 kg	6 kg
Height	47 cm	50 cm	50 cm	62.5 cm
Head circumference	33 cm	34.5 cm	36.5 cm	40.5 cm
Developmental delay	Poor stimulation reaction and no crying	Dull face, poor response, no crying, and only grunts	Dull face and only a low roar	Dull face, no crying, no laugh, and a low roar
Motor dyspraxia	Hypertonicity in extremities (Grade I)	Hypertonicity in extremities and poor embrace reflex	Hypertonicity in upper extremities (Grade III), lower limbs (Grade II), and unable to raise the head	Unable to raise the head and reach unsupported sitting. Hypertonicity in extremities (upper limbs: Grade III; lower limbs: Grade II)
Oro-intestinal problems	No suck, swallow, and gag reflexes	Uncoordinated sucking and swallowing and most of the milk needs to be nasally fed	Feeding difficulties, constipation, and gastroesophageal reflux	Enhanced feeding capacity and improved coordinated sucking and swallowing. Voluntary voiding and defecation
Cardiac malformations	Atrial septal defect 3.0 mm, patent ductus arteriosus, and moderate pulmonary hypertension	Atrial septal defect 2.5 mm, patent ductus arteriosus, pulmonary hypertension, precardiac region, and ii/6 systolic murmurs	Atrial septal defect 3.2 mm and pulmonary hypertension (40 mmHg)	ND
Facial features	ND	Bilateral temporal stenosis, thin upper lip, and large low set posteriorly ears	Bilateral temporal stenosis, thin upper lip, tent-like mouth, and large low set posteriorly ears	Bilateral temporal stenosis, thin upper lip, and large low set posteriorly ears
Skull and brain abnormalities	Skull flat	Skull flat	Skull flat	Skull flat and anterior fontanelle 1 cm × 1 cm
Other clinical features and clinical guidelines	Undescended testicles	Hydrocele of tunica vaginalis	Obviously pale trunk and limbs skin, fairly white skin of hands and feet, slightly cool, and small bilateral testicles	Hospitalized pediatrics with pneumonia (recurrent respiratory infections)

ND, not defined.

After admission, he was mechanically ventilated, treated for mild hypothermia and infection, and provided with other symptomatic support. However, during the treatment, he had a poor response, low limb muscle strength, and weak sucking and swallowing function and needed nasal feeding. Due to weak spontaneous breathing, it was difficult to remove the machine. When 3 weeks old, he was changed to noninvasive auxiliary ventilation, and no seizures occurred.

**FIGURE 1:**
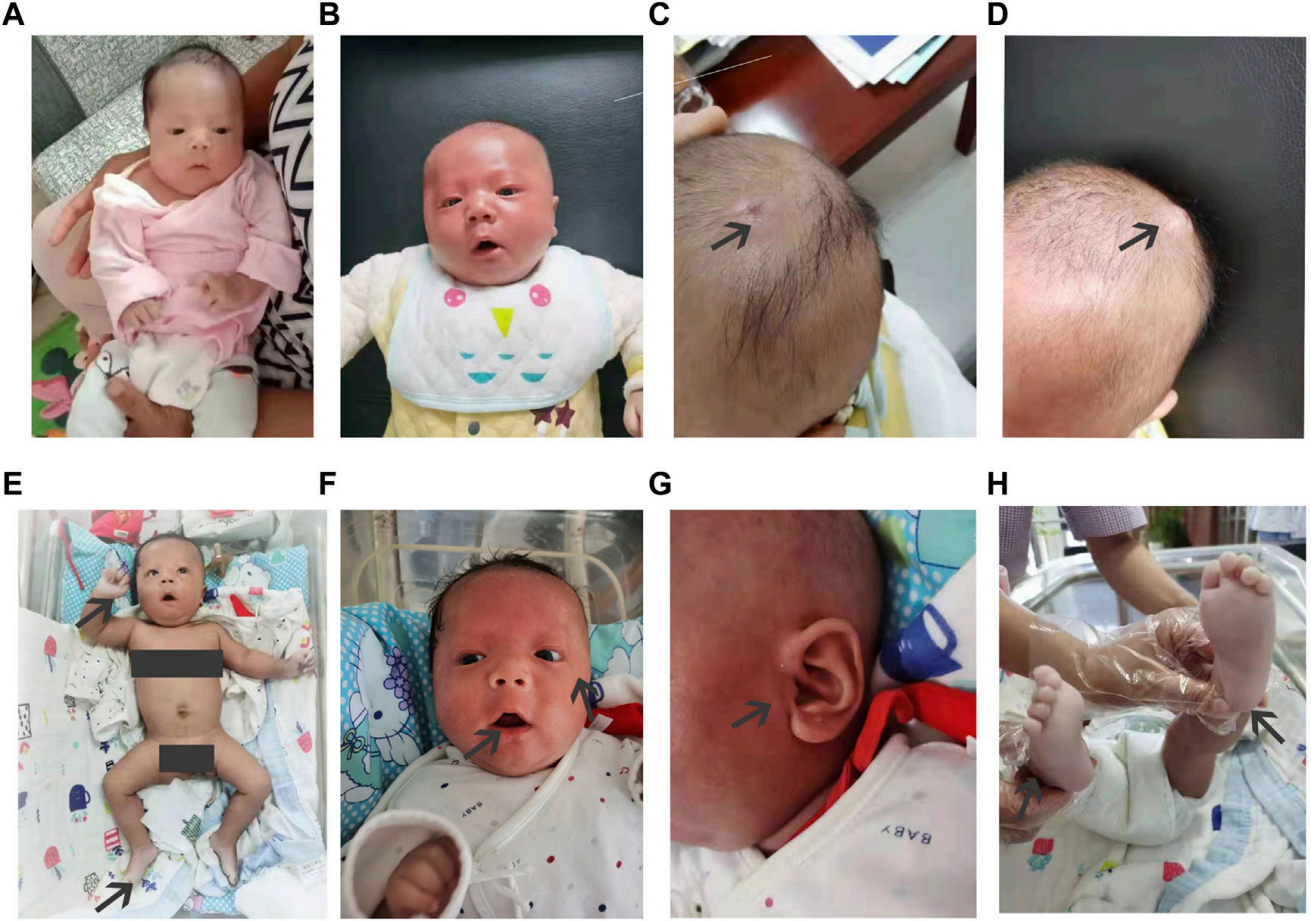
Clinical features of the infant from birth to when 17 weeks old. The newborn at 4 weeks **(A)**, 10 weeks **(B–E)**, and 17 weeks **(F–H)**. The infant shows characteristic features of Arboleda−Tham syndrome, e.g., broad nasal tip, thin upper lip, and tented mouth. A hemangioma was on the top of his head in the 10th week. Hands and feet skin color was fair and slightly cool in the 17th week.

At 4 weeks, the ventilator was withdrawn due to increased spontaneous breathing. His developmental delay remained evident. A head CT indicated neonatal hypoxic-ischemic encephalopathy, and the electroencephalogram presented bilateral asymmetric brain development and brain nerve cell damage. The subject had a dull face, bilateral temporal stenosis, thin upper lip, and large low set posteriorly ears and did not cry (Figure 1A). His muscle tone was in the extremities and his hug reflexes were poor. His sucking and swallowing were uncoordinated, and he received most of his milk *via* nasal feeding. The atrial septal defect was reduced to 2.5 mm with patent ductus arteriosus, pulmonary hypertension, precardiac region, and ii/6 systolic murmurs. Bilateral testicular B-ultrasound showed a left inguinal region; cryptorchidism was considered; and the right testicle was not detected (Table 1). Mouse growth factor was used to nourish the brain nerves with three courses of 30 µg by intramuscular injection once every other day (QOD) for 10 days. He also received rehabilitation such as touching, kneading the back, passive movement of limbs, audio−visual stimulation, and so on.

At 10 weeks, the infant only made a low roar, and feeding remained difficult with constipation and gastroesophageal reflux. He could not raise his head, and hypertonicity in the upper extremities was grade III and grade II in the lower limbs. His atrial septal defect was 3.2 mm, pulmonary hypertension was 40 mmHg, and hands and feet were obviously pale (Figures 1B–E). The baby also had small bilateral testicles.

At 17 weeks, he weighed 6.0 kg, measured 62.5 cm, and had a cranial circumference of 40.5 cm (<P_3_). The infant did not cry or laugh and only made a low roar. He was still unable to raise his head and sit without support, and failed to reach for subjects. Hypertonicity in his upper limbs was grade III and grade II in the lower limbs. His feeding capacity was enhanced, and his coordination of sucking and swallowing had improved (Table 1). He could voluntarily void and defecate (Figures 1F–H). According to the clinical phenotypes of the proband, the patient may have ARTHS.

### Clinical symptoms of other family members

**FIGURE 2:**
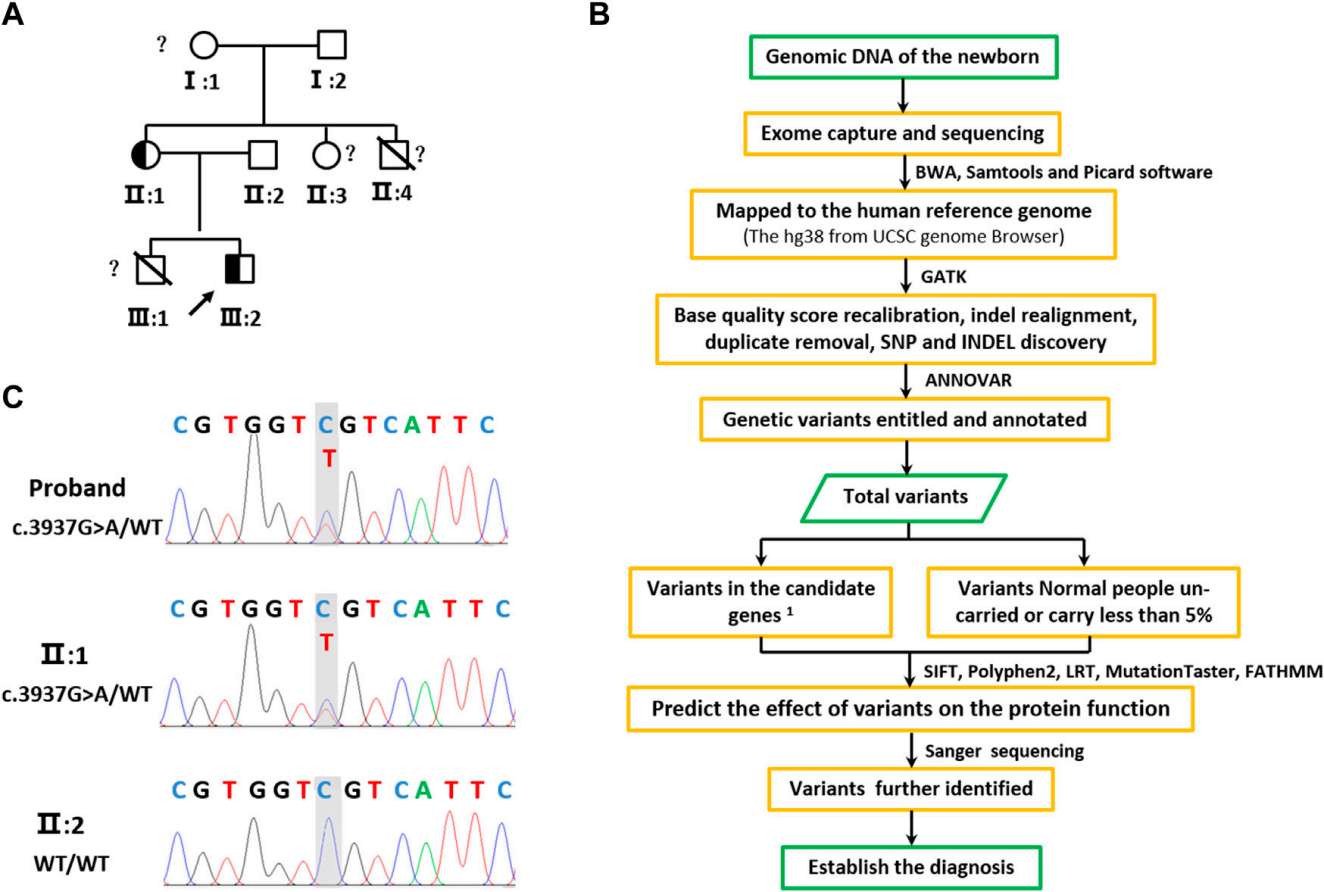
Pedigree tree and identified of KAT6A c.3937G>A variation. **(A)** Family pedigree. Black symbols indicate mutation carriers, while the other symbols indicate normal carriers. The arrow indicates the family proband. The peripheral blood genomic DNA of II:2 was used for WES. The peripheral blood genomic DNA of individuals II:1, II:2, and III:2 was used for Sanger sequencing. **(B)** Sequencing of the heterozygous variation c.3937G>A in the KAT6A gene of family members II:1, II:2, and the proband (III:1). Genome-wide exon sequencing and variants screening 1based on the disease and/or phenotypes and also 2based on the reports in OMIM, HGMD, and ClinVar.

The infant was the second child of his parents, and the first child (III:1) was a boy who died at about 3 months (Figure 2A). He was born at 37 + 2 weeks because of a decreased fetal heart rate. At birth, he weighed 3.250 g with normal amniotic fluid, low Apgar score, soft limbs, atrial septal defect (3 mm), and unexplained progressive aggravation of respiratory distress in the first hours after birth. He was assisted by mechanical ventilation through endotracheal intubation for 1 month and mainly fed by nasal feeding until 2 months old. After 2 months, he sounded a low roar; his upper limbs could move autonomously, but he could not move his legs; and his muscle strength was significantly reduced. At about 3 months, he died of asphyxia due to milk reflux aspiration at night, so his DNA was unavailable. According to his parents, he presented with a dull face, cape mouth, and large ears.

**FIGURE 3:**
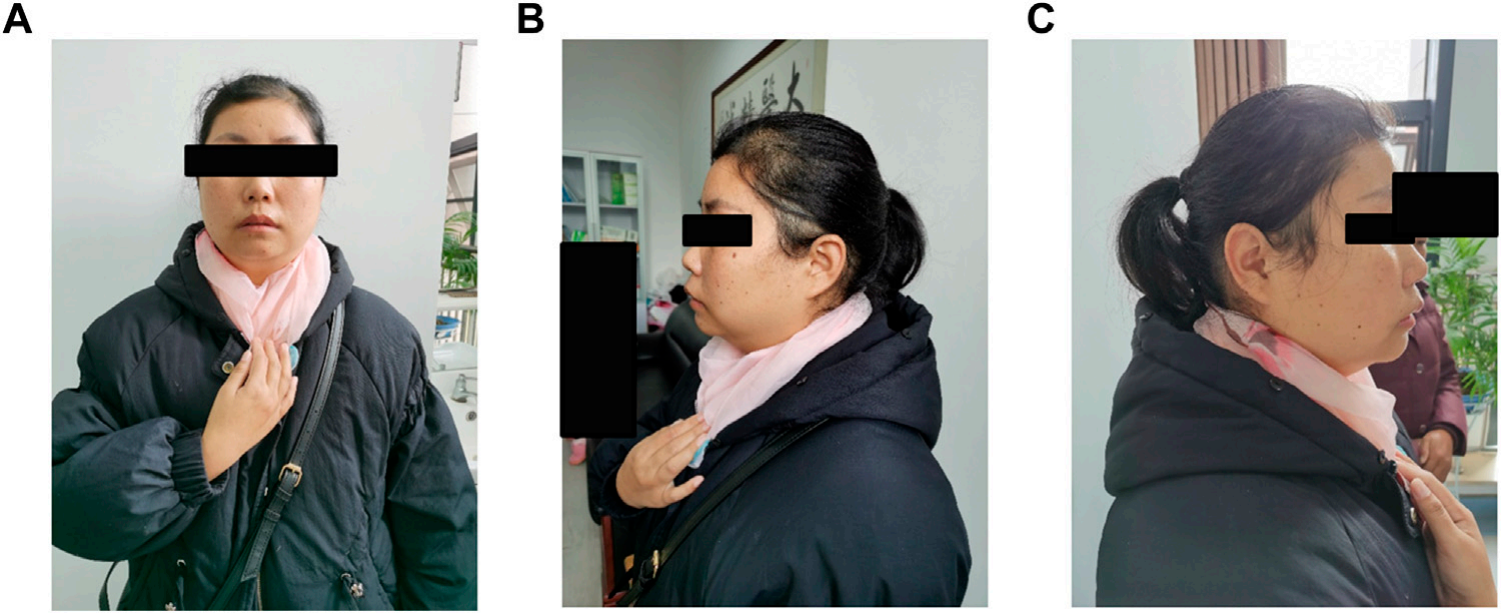
**(A–C)** Photos of the proband's mother. Physical characteristic features of this syndrome, such as bitemporal narrowing, broad nasal tip, low set ears, thin upper lip, micrognathia, and smooth philtrum are not observed.

II:1 was a 30-year-old female with slight cognitive impairment and expressive language disorder; nonverbal communication was better than verbal communication (Figure 3). II:3 was 26 years old, also with moderate cognitive impairment and expressive language disorder (Figure 2A). Their parents denied that either had other abnormalities other than intelligence. II:4 was a boy born over 30 years ago and had died 2 days after his birth without definitive pathogenesis; other growth and development defects were unclear. The grandparents of the infant presented no obvious clinical manifestations.

### Mutation c.3937G > A (p.Asp1313Asn) in *KAT6A* causing Arboleda−Tham syndrome

There are few reports of newborns with ARTHS, so WES was performed to detect the pathogenic variant (Figure 2B). WES produced about 2.98 MB sequences, and 99.71% of the exome was covered at least 10 times. Filtering of common sequence changes resulted in 250,875 variants, of which 6 variants in *PHKA2*, *TTN*, *CACNA1S*, *KAT6A*, and *CUL7* were associated with the subject (Supplementary Table S1).

**FIGURE 4:**
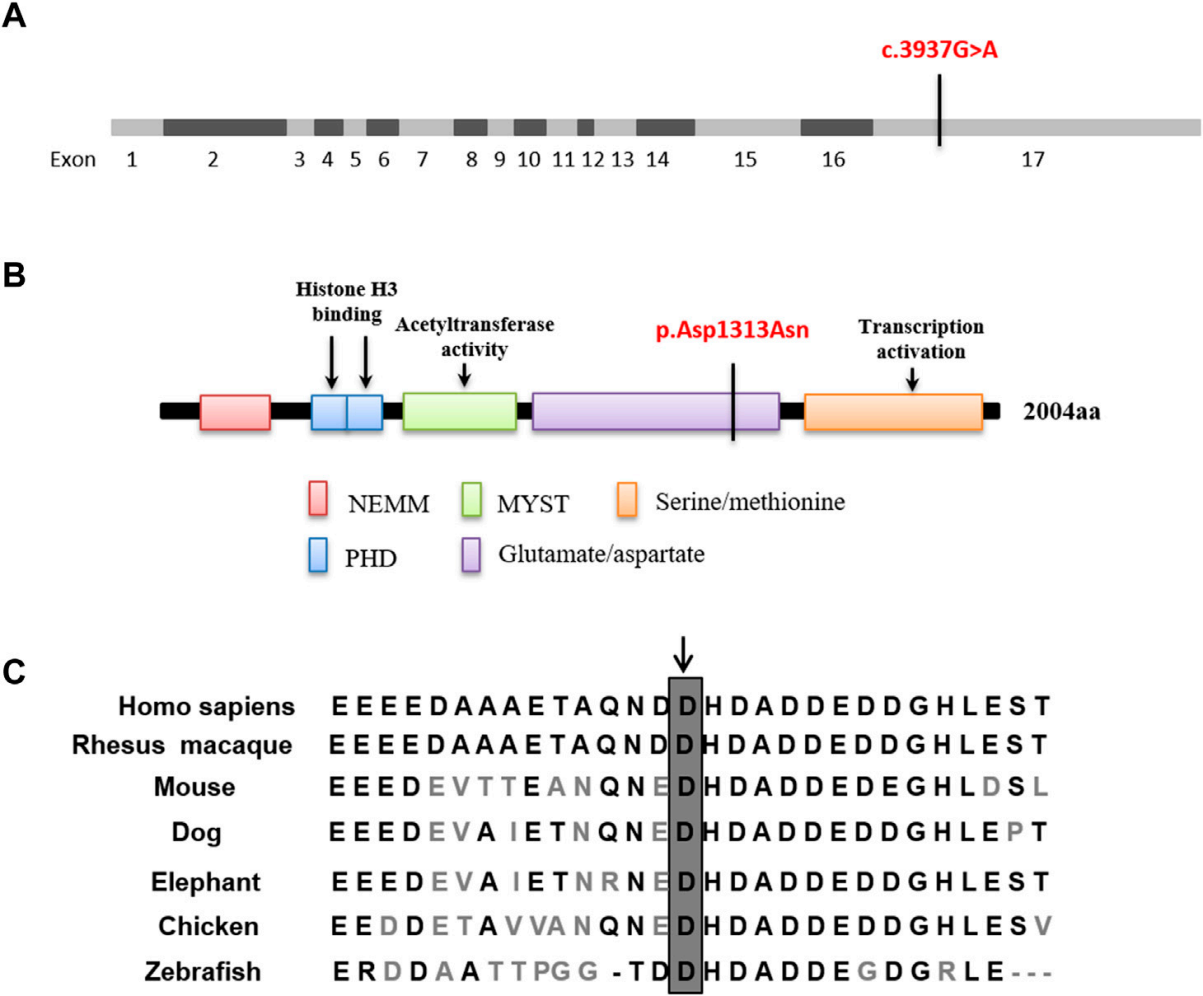
*In silico* analysis. **(A,B)** The *de novo* mutation c.3937G>A located in exon 17 which is predicted to lead to a missense mutation in the glutamate/aspartate domain. **(C)** Evolutionary conservation of the missense mutation. Sequence alignment of amino acids reveals that the amino acid at 1313 is highly conserved across different species.

The *de novo* c.3937G>A variant of the *KAT6A* gene (NM_006766.5) is located in exon 17, which changes aspartic acid (Asp) to asparagine (Asn) at codon 1313 (Figures 4A,B). This variant was confirmed by Sanger sequencing (Supplementary Table S2) and was absent in 100 healthy individuals. Furthermore, Sanger studies showed that the newborn’s mother carried this variant, but it was not detected in the father (Figure 2C). Evolutionary conservation analysis of the amino acid sequences of KAT6A proteins from different species shows that the impaired amino acid residue is highly evolutionarily conserved (Figure 4C), and SIFT (http://sift-dna.org) analysis showed that the variant was detrimental. The clinical features of the newborn were similar to ARTHS, so it was concluded that this *de novo* variant was the cause of this disease in the present family.

## Discussion

ARTHS manifests as a developmental delay, with about 90 patients being reported within 7 years. This case report describes the clinical features and treatment of one neonate with ARTHS from birth to when 17 weeks old. The main symptoms of ARTHS at birth were asphyxia, involuntary breathing, low muscle tone, early feeding, movement difficulties, weak crying, weakened muscle tone of the limbs, and embrace reflex. There was obvious developmental delay, as well as hypotonic and oro-intestinal problems in the first few months after birth (Table 1). Notably, the facial features were not obvious at birth.

Most neonatal genetic diseases have an early onset and progress rapidly with heterogeneous symptoms. Common manifestations include low muscle tone, feeding difficulty, respiratory failure, developmental delay, epilepsy, etc., therefore early diagnosis is difficult. The symptoms presented in this study will expand our understanding of the neonatal symptoms of ARTHS and provide a useful reference for clinical treatment.

At present, ARTHS cases are sporadic or inherited in autosomal dominant ways (Arboleda et al., 2015; Tham et al., 2015; Satoh et al., 2017; Bscch et al., 2019). In this study, the analysis of parental genotypes indicated that KAT6A c.3937G>A (p.Asp1313Asn) was transmitted by the newborn’s mother, but the mother was only mildly retarded (Figure 2C). There were two boys in the family who had died within 3 months of their birth, but the cause of their death was unclear, and III:1 presented ARTHS facial symptoms (Figure 2A). They were considered ARTHS, but detecting their genotypes and confirming this diagnosis was impossible. However, these results suggest that ARTHS could be inherited through incomplete penetrance.

KAT6 catalyzes the acetylation of lysine residues on histones and nonhistone proteins, playing a key role in regulating gene expression *via* modification of histone lysine residues that modulate chromatin organization. As an epigenetic modifier, the effect of pathogenic KAT6 variants is probably affected by both the genetic background and different environmental conditions, such as the abundance of acyl-CoAs and the metabolic status of the cell (Johayra et al., 2017; Voss and Thomas, 2018). This may be why there are currently no typical ARTHS symptoms in this family. The phenotypic characteristics such as developmental delay and ID, facial dysmorphism, speech delay, hypotonia, and cardiac defects are common in patients with ARTHS. There is also phenotypic variability between individual patients and rare features in a few patients affected by other variations apart from the KAT6A mutation. KAT6 deficiency or KAT6A harboring loss-of-function mutations regulates various developmental processes in mice and zebrafish. Homozygous deletion of the KAT6A gene in mice has resulted in high penetrance of ventricular septal defects. Ventricular septal defects and other congenital heart abnormalities are present in more than 50% of individuals with inactivating mutations in the KAT6A or KAT6B genes (Tham et al., 2015).

In summary, this is the first report to describe the clinical symptoms of a newborn with KAT6A mutation in the first months after birth and adds to the patient clinical data of ARTHS, especially symptoms in newborns. Also, the disease may be inherited through incomplete penetrance, so it should be considered cautiously.

## Data Availability

All datasets generated for this study are included in the article/supplementary material.
