# Supplementary material for: Clinical manifestations and genetic analysis of a newborn with Arboleda−Tham syndrome
Source: Front Genet. 2022 Oct 25;13:990098. doi: 10.3389/fgene.2022.990098 (PMC9641261; doi:10.3389/fgene.2022.990098)
Supplement: Supplementary file 1 [file Table1.docx]

| **Supplementary Table 1. Bioinformatics Analysis of the Whole-exome Sequencing Data** | |
| --- | --- |
| Number of bases mapped to target region (MB) | 2.98MB |
| Coverage of target region 1x (%) | 99.74 |
| Coverage of target region with at least 10x (%) | 99.71 |
| Coverage of target region with at least 20x (%) | 99.65 |
| Coverage of target region with at least 30x (%) | 99.49 |
| Coverage of target region with at least 50x (%) | 98.34 |
| Total variants | 250875 |
| Variants absent in the dbSNP137、HGMD、ClinVar database and predicting not benign | 3111 |
| variants with MAF^a^ <0.01 | 1189 |
| Nonsynonymous, splicing and InDel variants | 855 |
| Above variants maybe associated with some patients' clinical phenotypes | 6 |

**^a.^MAF, minor allele frequency**
