# Supplementary material for: Clinical manifestations and genetic analysis of a newborn with Arboleda−Tham syndrome
Source: Front Genet. 2022 Oct 25;13:990098. doi: 10.3389/fgene.2022.990098 (PMC9641261; doi:10.3389/fgene.2022.990098)
Supplement: Supplementary file 2 [file Table2.docx]

**Supplementary Table 2. Primers Used for *KAT6* mutation screening**

| Name | Foward Primer (5’-3’) | Reverse Primer (5’-3’) |
| --- | --- | --- |
| Exon2 | TCCATGGTGTAAAACTTACTCAGC | CAGAAATGTGAATTTGTAGCATCC |
| Exon 3 | TCACCTACTTAGCTGGAATTTAGG | CATACTTTCCGCTTGCCTTC |
| Exon 4 | TGGTGCATCTTGCTTTATTTG | TGAAAATGCTGGTCGTACATTC |
| Exon 5 | CCTGAAGCTTTCTAGGTTTTATGC | TGTGCTTTCACTAACATCCTCC |
| Exon 6 | AGATCCTGACTTAAGACTATCTGAAAC | AAAGTGCCAGATATGACAATGG |
| Exon 7 | AGGCTTCCTGGCAATAGATG | TCCTGCCACAGTTATCACCTC |
| Exon 8 | CATTCTTCTAAGAGTCAGTTTAATG | GCTGTTTCTCAGCCATGAAC |
| Exon 9 | CCTGAGAAGACAACATTTAGATTCAG | TCAAGATCCCTTCCAGTTCC |
| Exon 10 | TGTTACCTGGGTAGTAATGAGAGG | AGATGCAATATAGGTGGCCC |
| Exon 11 | TGGTCATGGACACCTTCTCC | CCGAGTGAGAACCAGCAAAG |
| Exon 12 | AGGGCTGTGGGTTTTGG | CACACACACAGAGAAGGTCCAC |
| Exon 13 | TTCTTGGTTGTAGAAGAGGGG | TGCCCTTTCATCAATAATCTG |
| Exon 14 | CTTACATGCTTCACCAGGGG | TCTCTACCACTGAAATAAAACTCTTG |
| Exon 15 | AAAAGGTCAGAATAGGTAAATGTAAG | AACGAGAAGGTGTAAGATAAACTGG |
| Exon 16 | TTTTGGCAAACAACATCCTC | AGGGTCTGTCACTGCTACTGC |
| Exon 17-1 | AAGAAGGAACTAGAAAATGTAAGACC | TTTCCCTACTCTTCTGCATATTAGC |
| Exon 17-2 | AGAGGAGCAGAGGCAGTCAG | CTGTGGAGGCGGTGGTG |
| Exon 17-3 | GATCCCTGTCCGCACCC | GGTTCATGGTAGTGGATGCC |
| Exon 17-4 | TGACCAACACCATTATGGACC | TGGTTTGTCAGTATAAAAGGTTCC |
